# Establishment of medium for laboratory cultivation and maintenance of *Fredericella sultana* for *in vivo* experiments with *Tetracapsuloides bryosalmonae* (Myxozoa)

**DOI:** 10.1111/j.1365-2761.2012.01440.x

**Published:** 2012-11-05

**Authors:** G Kumar, A Abd-Elfattah, H Soliman, M El-Matbouli

**Affiliations:** Clinical Division of Fish Medicine, Department for Farm Animals and Veterinary Public Health, University of Veterinary MedicineVienna, Austria

**Keywords:** bryozoan, cultivation, *Fredericella sultana*, maintenance, medium, *Tetracapsuloides bryosalmonae*

## Abstract

The freshwater bryozoan *Fredericella sultana* (Blumenbach) is the most common invertebrate host of the myxozoan parasite *Tetracapsuloides bryosalmonae*, the causative agent of proliferative kidney disease in salmonid fish. Culture media play an important role in hatching of statoblasts and maintaining clean bryozoan colonies for Malacosporea research. We developed a novel culture medium, Bryozoan Medium C (BMC), for the cultivation and maintenance of *F*. *sultana* under laboratory conditions. Statoblasts of *F. sultana* were successfully hatched to produce transparent-walled, specific pathogen-free (SPF) colonies that were maintained >12 months in BMC at pH 6.65. *Tetracapsuloides bryosalmonae* was successfully transmitted from infected brown trout, *Salmo trutta *L., to newly hatched *F. sultana* colonies in BMC, then from the infected bryozoan to SPF brown trout. This study demonstrated the utility of BMC (pH 6.65) for hatching statoblasts, long-term cultivation of clean and transparent bryozoan colonies and maintenance of the *Tetracapsuloides bryosalmonae* life cycle in the laboratory for molecular genetic research and other studies such as host–parasiteinteraction.

## Introduction

Members of the Phylum Bryozoa are generally small, sessile invertebrates that live on submerged surfaces, with most freshwater bryozoans grouped in the class Phylactolaemata (Wood *et al*. 1998). During warm months, bryozoans are found in almost any lake or stream that have suitable attachment sites, such as tree branches, roots, rocks, pilings or docks (Wood 2009). Bryozoans are typically colonial, consisting of connected zooid subunits, each of which has its own independent tentacular lophophore mouth, gut, muscles, nervous and reproductive systems, but adjacent zooids share certain tissues and fluids that physiologically unify bryozoan colonies (Wood *et al*. 1998; Wood & Okamura 2005). Bryozoans feed by filtering water for organic particles, small microorganisms such as diatoms and other unicellular algae that they capture with ciliated tentacles (Wood *et al*. 1998). Colonies typically reproduce asexually, through two routes: colony fragmentation or statoblast formation. Colony fragments can adhere to new substrates and grow to form new colonies (Buchsbaum *et al*. 1987). Bryozoans of the class Phylactolaemata can form encapsulated, seed-like statoblasts, which remain dormant and can withstand drying and freezing; when conditions are favourable, the statoblasts germinate to form a new colony (Wood & Okamura 2005).

One of the most common freshwater bryozoans is *Fredericella sultana*, which grows by budding zooids that form tubular and branching colonies (Okamura & Wood 2002). In addition to reproduction via statoblasts, *F. sultana* can undergo sexual reproduction and produces larvae in early summer (Tops, Hartikainen & Okamura 2009). *Fredericella sultana* colonies grow rapidly during spring, form large colonies by summer and overwinter either as living colonies or statoblasts (Wood 1973; Bushnell & Rao 1974; Tops *et al*. 2004; Morris & Adams 2006). The colonies have a tough, chitinous outer wall and are attached to submerged surfaces (Wood & Okamura 2005). Generally, field-collected bryozoans colonies are encrusted with opaque, inorganic particles in the body wall and sometimes associate with numerous ciliates, which hinder examination of developing parasite stages within the bryozoans (Pennak 1989; Morris & Adams 2006; Hartikainen & Okamaura 2012). Clean and ciliate-free bryozoan colonies are very important to examine the malacosporean parasite developmental stages (Morris, Morris & Adams 2002).

Proliferative kidney disease (PKD) significantly affects both farmed and wild salmonid fish in Europe and North America, causes economic losses and endangers wild fish populations (Feist *et al*. 2001; El-Matbouli & Hoffmann 2002). PKD is caused by the myxozoan parasite *Tetracapsuloides bryosalmonae* (Myxozoa: Malacosporea). *Tetracapsuloides bryosalmonae* spores develop in the kidney tubules of infected fish are released via urine to infect bryozoan hosts (Morris & Adams 2006; Grabner & El-Matbouli 2008). The life cycle of *T. bryosalmonae* requires two hosts: a vertebrate (salmonid fish) and an invertebrate (freshwater bryozoan) (Canning *et al*. 2000; Feist *et al*. 2001; Grabner & El-Matbouli 2008).

*Fredericella sultana* is the most common invertebrate host of *T. bryosalmonae* (Anderson, Canning & Okamura 1999; Feist *et al*. 2001; Grabner & El-Matbouli 2008). Within *F. sultana*, the parasite starts its development as single cells associated with the bryozoan body wall and then proliferates into spore-producing sacs in the body cavity; spores are released into the water, where they can infect fish and cause PKD (Longshaw *et al*. 2002; Tops & Okamura 2003; Morris & Adams 2006; Tops, Lockwood & Okamura 2006).

Bryozoan development is affected primarily by water temperature, pH, calcium and magnesium (Ricciardi & Reiswig 1994; Økland & Økland 2001); with lesser influence from potassium, sulphate and nitrate (Schopf & Manheim 1967; Økland & Økland 2001; Morris *et al*. 2002; Hartikainen *et al*. 2009). Several water chemistries and growth media have been used for short-term culture and maintenance of bryozoans in aquarium systems: artificial freshwater medium (0.35 mm CaSO_4_, 0.5 mm KCl, 0.5 mm MgSO_4_, 0.1 mm NaHCO_3_), Chalkley's medium (1.7 mm NaCl, 50 μm KCl, 50 μm CaCl_2_) and dechlorinated tap water (Wood 1996; Morris *et al*. 2002; Tops *et al*. 2004; McGurk *et al*. 2006; Grabner & El-Matbouli 2008).

Our objectives were to establish and optimize a medium for hatching *F. sultana* statoblasts, production of SPF colonies and long-term cultivation and maintenance of clean bryozoan colonies. We succeeded in reaching these goals and were able to establish the whole life cycle of *T. bryosalmonae* within a laboratory setting. Furthermore, application of optimized medium will facilitate a more detailed chronological study of parasite development in both hosts and collection of developmental stages of the parasite that are required for molecular genetic studies and other research activities.

## Materials and methods

### Collection of colonies and preparation of the culturing system

*Fredericella sultana* colonies were collected in April 2011 from the Lohr River, Germany (50°01′08′′N; 09°32′43′′E) at depths of 20–100 cm attached to roots of alder trees. Colonies were placed in containers filled with aerated river water and transported to the laboratory at the Clinical Division of Fish Medicine, University of Veterinary Medicine, Vienna, Austria.

A range of media were prepared (A–D, Table 1): each had different concentrations of calcium chloride dihydrate (CaCl_2_ H_2_O), magnesium chloride hexahydrate (MgCl_2_ 6H_2_O), potassium nitrate (KNO_3_), sodium bicarbonate (NaHCO_3_), magnesium sulphate heptahydrate (MgSO_4_ 7H_2_O) and sodium chloride (NaCl) and each at different pH (4.0, 5.65, 6.65, 7.65 and 9.0). Media were aerated overnight before use. Concentrated (100×) stock solutions of medium C (Bryozoan Medium C ‘BMC’) components were prepared in sterile 500-mL bottles (Table 2) and stored at 4 °C. Working solutions of BMC were prepared by adding 10 mL of each of the six stock component solutions to 9.940 L Milli-Q water, and the final solution mixed thoroughly.

**Table I:** Component concentrations of the four media

Components	Concentrations of media (L^−1^)			
Medium A (mm)	Medium B (mm)	Medium C (mm)	Medium D (mm)
CaCl_2_·H_2_O	1.0	0.5	0.1	0.025
MgCl_2_·6H_2_O	0.2	0.1	0.05	0.025
KNO_3_	0.06	0.03	0.03	0.0075
NaHCO_3_	0.5	0.25	0.25	0.125
MgSO_4_·7H_2_O	0.08	0.04	0.04	0.02
NaCl	1.7	1.7	1.7	0.85

**Table 2:** Component recipe for 100× stock solution of Bryozoan Medium C

Components	Formula weight	Final concentration in stock solution (m)	Grams for 500 mL of 100× stock solution (g)
CaCl_2_·H_2_O	147.02	0.1	7.35
MgCl_2_·6H_2_O	203.31	0.05	5.08
KNO_3_	101.11	0.03	1.516
NaHCO_3_	84.01	0.25	10.50
MgSO_4_·7H_2_O	246.48	0.04	4.92
NaCl	58.44	1.7	49.67

### Statoblast hatching experiments

Free statoblasts were dissected from *F. sultana* colonies with fine needles and forceps, collected by pipette and pooled into a single container. Media (A–D) were tested at the 5 pH values (4.0, 5.65, 6.65, 7.65 and 9.0), in duplicate, in a total of 40–12 cm plastic Petri-plates, each of which contained 30 statoblasts. All plates were stored at 4 °C for 2 weeks in the dark and subsequently incubated at 15 °C for a week. After attachment of statoblasts to the dish-surface, each pair of plates was transferred to 2-L containers filled with the same medium, at the same pH, and held at 18 °C with slow aeration and illumination. After statoblasts hatched, three punctures were made in each plate to enhance circulation of the medium, and the plate placed upside down in racks, back in the same containers.

We compared statoblast hatch rates of our media with Chalkley's medium and dechlorinated tap water, which were prepared according to the methods of McGurk *et al*. (2006) and Grabner & El-Matbouli (2008), with statoblasts incubated and maintained as for our media.

### Colony cultivation experiments

Small pieces (3–4) of alder tree roots with mature *F. sultana* colonies were glued to each of 20–12 cm plastic Petri-dishes. The dishes were then held singly in 5-L containers, each of which contained one of the four media, at one of the five pH values, and held at 18 °C with slow aeration and illumination. Growth in the different media was compared with *F. sultana* colonies held in Chalkley's medium or 1-week old aerated dechlorinated tap water (maintained as for the test media).

### Maintenance of colonies

Bryozoans, both from fresh hatched statoblasts and mature colonies, were maintained according to Grabner & El-Matbouli (2008). Briefly, 5 algal species (*Cryptomonas ovata*,* Cryptomonas* sp., *Chlamydomonas* sp*., Chlamydomonas reinhardii*,* Synechococcus* sp.) were cultured in sterile Guillard's WC-Medium. Approximately, 50 mL of *C. ovata* and 12 mL of the other four algae were added to the 2-L hatching experiments, every second day. Similarly, 125 mL of *C. ovata* and 30 mL of the other four algae were added to the 5-L mature colony experiments, every second day. The total medium of each container was replaced weekly with fresh medium. Bryozoan colonies were observed daily using a dissecting microscope.

### *Tetracapsuloides bryosalmonae* life cycle experiments

BMC (pH 6.65) was used for the *T. bryosalmonae* life cycle experiment, as it gave best results for statoblast hatching and colony maintenance (see Results).

*Fredericella sultana* colonies naturally infected with *T. bryosalmonae* were collected from the Lohr River, Germany and placed in 8-L aquaria with BMC (pH 6.65) and held at 18 °C. Three SPF brown trout (4–5 cm long) were cohabitated for 8 h per day for 14 days with the infected *F. sultana* colonies. During this period, bryozoan colonies were fed daily as previously mentioned. After infection, fish were placed in 20-L aquaria with flow-through water.

Eight weeks post-infection, the three infected brown trout were cohabitated for 8 h per day for 14 days with laboratory-hatched, 2-month-old SPF *F. sultana* in an 8-L aquarium filled with BMC (pH 6.65) at 18 °C. After 8-h cohabitation, fish were transferred to a 10-L aquarium with spring water at the same temperature and fed with salmonid feed. The bryozoan colonies were fed with algae as mentioned above, and the entire medium replaced once per week.

## Results

### Statoblast hatching

**Figure 1:**
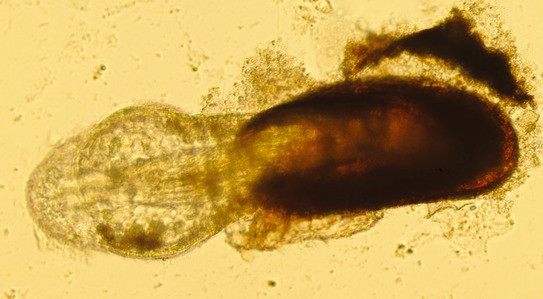
Three-week-old *Fredericella sultana* zooid, hatched in the laboratory in bryozoan medium C (pH 6.65) (x100).

Table 3 shows the number of the 30 statoblasts that hatched in each of the four media tested, at each of five pH values. Medium BMC at pH 6.65 produced the best hatching result: 8–10 statoblasts/plate (33%) hatched (Fig 1) after 2 weeks of incubation. Fewer hatched in BMC at pH 5.65 or 7.65, after 4 weeks, or 5–6 weeks in medium D at pH 6.65. No statoblasts hatched in media C and D at pH 4.0 or 9.0, or in media A and B at any pH. The pH of BMC (pH 6.65) was not significantly (+0.08) affected by using cultured algae species for feeding bryozoans. Hatched statoblasts started to colonize onto Petri dish plates and proliferated into zooids. Initially, hatched colonies developed 2–3 zooids. Six weeks post-hatching; colonies were growing at a rate of 12–14 zooids per month and produced statoblasts. We maintained the hatched colonies in BMC (pH 6.65) for more than 1 year.

**Table 3:** Hatching rate of *Fredericella sultana* statoblasts in media (A–D) at different pH values. Each trial run with duplicate plates

Media	Hatching rate of *Fredericella sultana* statoblasts (number of hatched statoblasts/total number of statoblasts)										
pH 4.0		pH 5.65		pH 6.65		pH 7.65		pH 9.0	
Plate 1	Plate 2	Plate 1	Plate 2	Plate 1	Plate 2	Plate 1	Plate 2	Plate 1	Plate 2
A	NH	NH	NH	NH	NH	NH	NH	NH	NH	NH
B	NH	NH	NH	NH	NH	NH	NH	NH	NH	NH
C	NH	NH	1/30	2/30	8/30	10/30	2/30	1/30	NH	NH
D	NH	NH	0/30	1/30	1/30	1/30	1/30	0/30	NH	NH

NH, not hatched.

### Viability and maintenance of mature *Fredericella sultana* colonies

**Figure 2:**
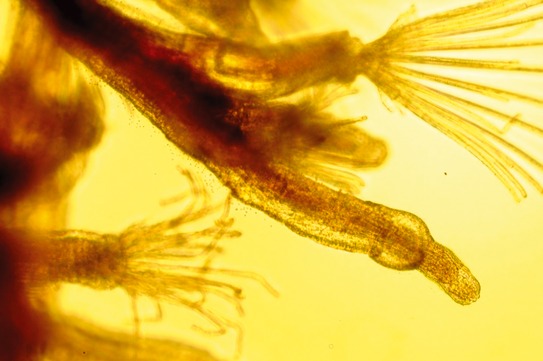
Clean and transparent laboratory-cultured *Fredericella sultana* colony, showing tentacles (x40).

Table 4 shows the survival and growth of mature *F. sultana* colonies under the different media and pH conditions. BMC at pH 6.65 gave the best results, and colonies survived and thrived for >12 months. Ciliates such as *Vorticella* species and *Carchesium* species were found initially on field-collected *F. sultana* colonies; however, those colonies were ciliate-free after 2 weeks of incubation in BMC (pH 6.65). The colonies were clean and transparent (Fig 2), proliferated and started to make new zooids. Colonies grew 24–31 zooids per month and produced statoblasts.

**Table 4:** Survival of *Fredericella sultana* colonies in media (A–D) at different pH values

Media	Time period of maintenance of mature *Fredericella sultana* colonies					
pH 4.0 (weeks)	pH 5.65 (months)	pH 6.65 (months)	pH 7.65 (months)	pH 9.0 (weeks)
A	4–5	2	2	2	4–5
B	4–5	2	3	2	4–5
C	4–5	4	>12*	3	4–5
D	4–5	2	2	2	4–5

* Colonies are still maintaining and growing in BMC under laboratory conditions until now.

In other media and pH, colonies could be maintained 3–4 months in BMC (pH 5.65 or 7.65) and 2 months in medium D (pH 5.65, 6.65, 7.65). While colonies survived 4–5 weeks at pH 4.0 or 9.0 in all media, they subsequently suffered massive mortalities. Survival of *F. sultana* colonies was poor (2–3 months) in media A or B at pH 5.65, 6.65 and 7.65.

### Hatching of statoblasts and maintenance of colonies in Chalkley's medium or in dechlorinated tap water

Of 30 statoblasts, 2–3 statoblasts hatched after 4–5 weeks of incubation in Chalkley's medium, while 1–2 statoblasts hatched after 6–7 weeks of incubation in dechlorinated tap water. Colonies survived in Chalkley's medium for up to 6 months and in dechlorinated tap water for up to 4–5 months.

### Infection of SPF *Fredericella sultana* colonies with *Tetracapsuloides bryosalmonae*

**Figure 3:**
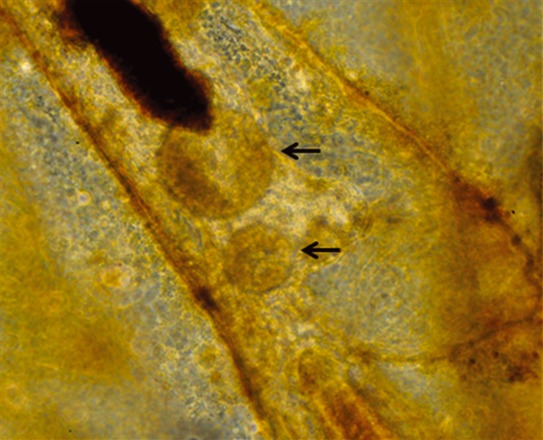
Two spore sacs of *Tetracapsuloides bryosalmonae* (arrows) within a laboratory-infected *Fredericella sultana* colony (x100).

Four weeks post-exposure to infected brown trout, free swirling spores were observed in the body cavity of SPF *F. sultana* colonies. Fifty per cent of the colonies were infected with *T. bryosalmonae*. During the following weeks, more zooids within the same colony of bryozoan showed overt infections with mature sacs (Fig 3), and spores were seen floating in the metacoel. Typically, 2–6 mature sacs were observed in zooids. Infected colonies did not produce any statoblasts.

## Discussion

Reliable cultivation and maintenance of *F. sultana* is regarded as an essential basis for establishment of the *T. bryosalmonae* life cycle in the laboratory (Grabner & El-Matbouli 2009). Different culture media have been used for maintenance of bryozoans: artificial freshwater medium, Chalkley's medium or freshwater protozoan culture medium and dechlorinated tap water (Morris *et al*. 2002; McGurk 2005; Grabner & El-Matbouli 2008). Alternatively, bryozoans can be cultivated using a recirculating aquarium ‘mesocosm’ culture system (Wood 1971, 1996), modified by Tops *et al*. (2004). However, these systems have disadvantages that include growth of chironomid larvae and ciliates, a requirement of natural water source to seed the tanks and the use of goldfish in the culture system. Low nutrient microcosms decrease the growth and increase the mortality of *F. sultana* colonies, and *vice versa* (Hartikainen *et al*. 2009). In the present study, we observed mortalities of *F. sultana* colonies in dechlorinated tap water, which we suspect is too mineral-poor for long-term maintenance of bryozoans. A clear requirement for strong colony growth is constant nutrient concentrations in the culture medium along with proper feeding of algae. Grabner & El-Matbouli (2008) used autoclaved mud from a fish pond to provide sediment-containing organic material to promote growth of bacteria and protozoa as nutrition for the bryozoa, whereas McGurk (2005) added higher concentrations of algae and protozoa directly to bryozoans. While both of these supplementation methods work, they have disadvantages that include accumulation of mud or algae particles on the surface of the bryozoans and the Petri dish plates, which increases ciliate accumulation and makes colonies less transparent.

In the present study, we fed bryozoans with cultured algae, grown in algal medium (CaCl_2_, MgSO_4_, NaHCO_3_, K_2_HPO_4_, NaNO_3_, Na_2_SiO_3_ and vitamins such as thimamine, biotin, cyanocobalamin). Introduction of these cultured algae did not show any adverse effects on the growth of colonies.

We also tested various component concentrations and pH in the medium in which the bryozoans were grown. Calcium and magnesium are known to be important components of natural water bodies that support bryozoans (Økland & Økland 2001). Calcium plays a pivotal role in the physiology and biochemistry of many cellular processes (Berridge, Bootman & Lipp 1998), and specifically, Ca^2+^ ions are essential components of the bryozoan body wall (Økland & Økland 2001; Wood 2009). Magnesium is an essential mineral for cell division, nucleic acids and protein synthesis (Maguire & Cowan 2002). Therefore, calcium and magnesium likely play an important role in statoblast hatching and colony growth.

*Fredericella sultana* naturally grows in the calcium and magnesium concentrations of 0.7–52.5 and 0.0–7.3 mg L^−1^, respectively (Økland & Økland 2001). We tested calcium concentrations of 147, 73.5, 14.7 and 3.65 mg L^−1^ and magnesium concentrations of 40, 20, 10 and 5 mg L^−1^ in media A, B, C and D, respectively. Statoblasts did not hatch in media A or B, while hatching rate was poor and slow in medium D. Hatching rate and speed was best in medium C (‘BMC’). We could not compare our hatching rate with previous studies, which used artificial freshwater medium and Chalkley's medium as these data were not recorded (Morris *et al*. 2002; McGurk *et al*. 2006). We found statoblast hatching rates were poor and slow in both Chalkley's medium and dechlorinated tap water, which suggests that both low and high component concentrations affect hatching of statoblasts.

Sodium chloride reduces the growth of protozoa such as ciliates (Ion *et al*. 2011). Ciliate contamination (*Rotaria* and *Vorticella* species) is among the problems in growing bryozoan colonies (Morris *et al*. 2002). We observed *Vorticella* species and *Carchesium* species, initially in our field-collected colonies. However, colonies became ciliate-free after incubating in BMC, which we suspect is a direct result of the presence of NaCl (100 mg L^−1^) in the BMC suppressing ciliate growth.

The pH is known to affect bryozoan growth: Økland & Økland (1986) demonstrated that acidic conditions have negative effects on bryozoa. We showed that pH played an important role in success of statoblast hatching and long-term maintenance of bryozoan colonies. Statoblasts did not hatch, and viability of colonies was not maintained in BMC at pH 4.0 or pH 9.0, showing that neither low nor high pH values are suitable for hatching statoblasts and growth of bryozoan colonies; moderately high or low pH (5.65, 7.65) had low hatching rates. We found that pH 6.65 BMC was optimum for hatching, viability and transparency of bryozoan colonies over the long term (>12 months).

We found that 33% of statoblasts had hatched after 2 weeks of incubation in BMC (pH 6.65). This rate was slower than the rate observed by Hartikainen *et al*. (2009), who report statoblasts hatched after 4 days of incubation in artificial pond water. We suspect that some untested factor such as age, development or environmental history or water chemistry may be the cause of slow hatching rate of statoblasts in BMC (Bushnell & Rao 1974). We determined there was no significance difference in the hatching rate of statoblasts that had been stored at 4 °C for 2 weeks or 5 weeks in the dark.

In BMC at pH 6.65, we observed a colony growth rate of 12–14 zooids per month, after 6 weeks post-hatching, with no colony mortality. This rate is slower than that reported by Hartikainen *et al*. (2009) who observed hatched colonies had developed 9–11 zooids after 15 days of incubation in artificial pond water, but with some mortality over 20 days, in high nutrient microcosms. The slower growth rate that we observed may be due to some insufficiency arising from the switch from a natural river environment to BMC and feeding with a limited set of algae species.

We consider that a hybrid approach may be best for growth and maintenance of bryozoans in the laboratory: a combination of the aquarium bryozoan culture system (goldfish system) and our clean BMC system. The goldfish system is suitable for generating large-scale bryozoan growth (Wood 1996; Tops *et al*. 2004; Hartikainen *et al*. 2009; Hartikainen & Okamaura 2012) but does not produce SPF bryozoans because of the use of natural pond water and gold fish. Our BMC system has lower bryozoan growth rates, but it is a clean system that produces SPF colonies essential for maintaining and studying malacosporean parasite life cycles in the laboratory. We showed that a system that contains BMC (pH 6.65) is suitable for transmission of *T. bryosalmonae* from infected brown trout to SPF *F. sultana* and supports the further development of the parasite in bryozoan colony.

In conclusion, we have shown that BMC (pH 6.65) can be used for rapid hatching of bryozoan statoblasts, long-term cultivation and maintenance of clean colonies. This facilitates the establishment of the *T. bryosalmonae* life cycle under laboratory-controlled conditions, to permit study of host–pathogen interaction and also collection of parasite developmental stages for molecular genetic studies and other research activities.
